# Prevalence of Human Bocavirus in Human Tonsils and Adenoids

**DOI:** 10.3201/eid1507.090102

**Published:** 2009-07

**Authors:** Nathalie Clément, Gino Battaglioli, Ryan L. Jensen, Bruce C. Schnepp, Philip R. Johnson, Kirsten St. George, R. Michael Linden

**Affiliations:** Mount Sinai School of Medicine, New York, New York, USA (N. Clément, R.M. Linden); Wadsworth Center, Albany, New York, USA (G. Battaglioli, K. St. George); Nationwide Children’s Hospital, Columbus, Ohio, USA (R.L. Jensen, B.C. Schnepp, P.R. Johnson); 1 Current affiliation: University of Florida, Gainesville, Florida, USA.; 2 Current affiliation: Children’s Hospital of Philadelphia, Philadelphia, Pennsylvania, USA.; 3 Current affiliation: King’s College, London, UK.

**Keywords:** Bocavirus, tonsillar tissue, viral persistence, pediatric, letter

**To the Editor:** Recently, Longtin et al. (*1*) reported a high rate (43%) of human bocavirus (HBoV) infection in a group of children chosen to serve as controls in a study of HBoV prevalence among hospitalized children and adults. In contrast, previous reports had found low HBoV prevalence rates of 0%–1% in control groups (*2**,**3*). Attempting to explain this surprising difference in rates, Lu et al. (*4*) suggested that selection of control patients may be related to the difference in rates. The control group used in the Longtin study were primarily (71%) children undergoing elective surgery; previous studies had selected control groups from other sources, including well children on routine visits and outpatients with nonrespiratory symptoms. Because the Longtin study surgeries were mainly tonsillectomies, adenoidectomies, and myringotomies, Lu et al. examined the possibility that tonsillar tissues, which include the adenoids, are sites of persistent HBoV infection. When these researchers extracted DNA from tonsillar lymphocytes obtained from pediatric patients who had undergone tonsillectomies or adenoidectomies, they detected HBoV DNA in 32% of the samples (*4*). These findings strongly suggest a connection between HBoV and tonsillar tissue. Therefore, we tested a number of tonsillar samples for the presence of HBoV DNA.

Sample acquisition was approved by the Nationwide Children’s Hospital Institutional Review Board. Tonsils and adenoids were obtained from 91 patients who underwent elective surgery at Nationwide Children’s Hospital from June through September 2004. Patients’ ages ranged from 1–16 years (median 5.9 years; age was unknown for 4 patients).

Samples consisted of surgically removed tonsil or adenoid tissues. DNA was extracted and its concentration was determined as previously described (*5*). Two primer sets were used for HBoV detection by using real-time PCR with SYBR Green detection and melting-point determination. We designed primers 3097F (5′-GTC-CAA-TTA-CAT-GAT-CAC-GCC-TAC-TC) and 3420R (5′-TGC-GTC-CAC-AGT-ATC-AGG-TTG-TTG) that targeted the viral protein ½ (VP1/VP2) region of HBoV. The nonstructural protein 1 (NP1) region was targeted by using primers 188F and 542R from Allander et al. (*6*) Each 20-μL reaction contained SYBR Green JumpStart Taq ReadyMix (Sigma, St. Louis, MO, USA), 4 mmol/L MgCl_2_, 250 nmol/L primers, double-distilled H_2_O, and 2 μL of DNA (50–200 ng) cycled on an ABI PRISM 7900HT (Applied Biosystems, Foster City, CA, USA) instrument at 94^o^C for 2 min, followed by 40 cycles of 94°C for 20 s, 60°C (NP1 primers) or 68°C (VP1/VP2 primers) for 20 s, and 72°C for 14 s. Amplification and melting curves were analyzed with 7900HT 2.2.1 software (Applied Biosystems); positive samples were verified by sequence analysis. Sequenced VP- and NP1-generated amplicons were 99%–100% identical to HBoV strain ST1 (*6*). The detection sensitivities of the VP and NP1 assays, determined by using a plasmid construct containing the full HBoV genome, were 1–5 and 5–10 gene copies/reaction, respectively.

Our testing identified HBoV DNA in 5 (5.5%) of the 91 children who underwent elective tonsillectomy/adenoidectomy. Ages ranged from 1.9–4.6 years, with a median age of 3.4 years. The reason for the much lower HBoV prevalence in this group of children, compared with prevalences found in studies by Longtin et al. (*1*) and Lu et al. (*4*), is unclear. Lu et al. (*4*) reported a much higher HBoV rate of lymphocytes from adenoids (56%) than from tonsils (16%). Although we did not know the exact tissue type of each sample, only that tonsils, adenoids, or both combined could be present, the 5.5% rate we found was about one third the rate found in tonsil lymphocytes and about one tenth the rate Lu et al. found in adenoid lymphocytes.

A seasonal effect may contribute to the large discrepancies found in HBoV prevalences. Apparently, viruses can persist in tonsillar tissue well after the symptomatic phase of illness. In children with no signs of acute respiratory infection, Drago et al. (*7*) reported that 45.5% of samples contained viral nucleic acid. Depending on the duration of persistence, asymptomatic children, sampled shortly after the season of the virus in question, would be more likely to have detectable virus in their tonsillar tissue. The Longtin study samples were collected from December through April; our study samples were collected from June through September. If HBoV is seasonal as has been suggested (*3*), it may have been circulating in the target population before samples were taken and persisted only in tonsillar tissues. Thus, if tonsillar tissue from asymptomatic children was obtained within the persistence period after the HBoV season, samples would be HBoV positive; those obtained shortly after the persistence period would have a much lower rate.

Differences in patient age in the 3 studies may also have contributed to the different rates observed. The Longtin group was substantially younger (median age, 23 months) than the Lu group (median, 5 years) or our group (median, 5.9 years). Preliminary seroepidemiology reports indicate the presence of HBoV antibodies in >50% of children by 2–3 years of age (*8**,**9*).

The detection of HBoV in the tonsillar tissues we tested showed a higher rate of infection than would be expected in an asymptomatic population. However, the rate was far lower than that previously reported for tonsillar tissues (*1*,*4*).
